# Pesticide Residues in Commonly Consumed Vegetables in Henan Province of China in 2020

**DOI:** 10.3389/fpubh.2022.901485

**Published:** 2022-06-10

**Authors:** Cuicui Ma, Dandan Wei, Pengling Liu, Keliang Fan, Luting Nie, Yu Song, Mian Wang, Lulu Wang, Qingqing Xu, Juan Wang, Jiayu Shi, Jintian Geng, Mengzhen Zhao, Zexin Jia, Changsheng Huan, Wenqian Huo, Chongjian Wang, Zhenxing Mao, Shan Huang, Xin Zeng

**Affiliations:** ^1^Department of Epidemiology and Biostatistics, College of Public Health, Zhengzhou University, Zhengzhou, China; ^2^Department of Occupational and Environmental Health Sciences, College of Public Health, Zhengzhou University, Zhengzhou, China; ^3^Institute for Special Food Inspection, Henan Province Food Inspection Research Institute, Zhengzhou, China; ^4^Department of Social Medicine, College of Public Health, Zhengzhou University, Zhengzhou, China

**Keywords:** vegetables, pesticides, residues, Henan province, GC-MS

## Abstract

**Background:**

Pesticides are widely used in agricultural production to control insect pests and regulate plant growth in China, which may result in the presence of some pesticide residues in the vegetables. However, few studies of monitoring pesticides have been conducted in Henan Province. The aim of this study was to evaluate the level of pesticide residues in commonly consumed vegetables in the regions of Henan Province.

**Methods:**

In this study, we collected 5,576 samples of 15 different vegetables in 17 areas from Henan Province during 2020. Eight kinds of pesticides were analyzed by gas chromatography-mass spectrometry (GC-MS), including procymidone, lambda-cyhalothrin, cypermethrin, pendimethalin, isocarbophos, isazophos, fenthion and deltamethrin. The chi-square test was used to compare the detection rates of pesticide residues in different regions.

**Results:**

Of all the pesticides above, procymidone, lambda-cyhalothrin, cypermethrin, pendimethalin and isocarbophos were detected in vegetables, the detection rates were 27.0%, 16.2%, 11.4%, 3.5%, and 1.9%, respectively. However, isazophos, fenthion, and deltamethrin were not detected. In addition, procymidone, lambda-cyhalothrin, and cypermethrin were detected in urban areas, while pendimethalin was detected in rural areas. The detection rates of cypermethrin and pendimethalin in rural were 19.8% and 5.4%, respectively, which in urban were at relatively lower levels (13.7% and 1.9%, respectively) (*P* < 0.05). Compared the differences of pesticide detection rates among five areas of Henan province, we found that there were statistical differences in the detection rates of procymidone, cypermethrin and lambda-cyhalothrin in different regions (all *P* < 0.05).

**Conclusion:**

The results have revealed that the pesticide residues are present. Higher detection rates and more types of pesticides were found in rural areas than urban areas. In addition, there were higher detection rates in Eastern Henan. The findings provided valuable information on the current pesticide residues status, which can be a reference of pesticide supervision and management.

## Introduction

Pesticides are widely used chemical agents which are used to control insect pests and regulate plant growth (1). Moreover, China is a large agricultural country, so pesticides play an important role in agricultural production. Although pesticides can improve the quality of agricultural products to a certain extent and bring some benefits to agricultural production, we also need to be aware of a series of problems caused by pesticides such as environmental pollution and health hazards (2). Pesticide residues are a serious problem due to their persistence, biological magnification and toxicity (3). Previous studies have shown that tea (4) and cooking oil (5) contain pesticide residues, which may pose potential hazards if eaten untreated. Studies have shown that the ingestion of food containing pesticide residues can cause cancer (6), heart disease (7) and other diseases to a certain extent. There is also a body of evidence that long-term pesticide exposure, even at low doses, can lead to negative effects such as birth defects, reduced birth weight, and fetal death (8). In addition, pesticide residues will also affect the export of agricultural products.

As the main source of our daily intake of vitamins and nutrients, vegetables are the important part of our daily life and are closely related to our health. In most Chinese people, each of us consumes at least 300 g of vegetables averagely per day (9). As people's living standards improve, the demand for vegetables is also increasing. In order to ensure the yield and quality of vegetables, farmers may spray pesticides in large doses during vegetable cultivation in order to prevent and control pests and diseases (10). And some farmers use pesticides incorrectly due to their lack of expertise in pesticide use and improper management of pesticides (11, 12), bringing about a series of pesticide residue problems. The problem of pesticide residues in vegetables is a global public health issue (13). With the growing population and accelerated urbanization, pesticide intake has generally increased in many countries around the world, and China is no exception. From 2015 to 2019, China ranked first in the world in terms of pesticide consumption, followed by the United States, which is a matter of concern (14).

The detection of pesticide residues in vegetables has always been a great challenge, because the composition of target substances is complex and we may can't achieve the expected detection effect due to the interference of multiple matrix substances (15). As an accurate qualitative and quantitative method, gas chromatography-mass spectrometry (GC-MS) is widely used in pesticide detection. Based on abundant advantages of GC-MS, such as fast, simple, sensitive, accurate, and good selectivity (16, 17), it can accurately identify target analytes and perform quantitative analysis, making it a highly sensitive and selective method.

The World Health Organization stressed that pesticide residues are a priority issue and it states that the effects of pesticides on human health are not immediate but long-term (18). At present, pesticide exposure in China is a problem that should be paid attention to. Pesticide has been detected in dust (19), river (20), drinking water (21), breast milk (22), dietary (23), and pediatric (24) and adult (25). In addition to the acute poisonings associated with pesticides including dizziness and headache, skin allergies, and burning of eyes (26), chronic hazards associated with long-term exposure are also a problem that cannot be ignored such as cancers, Alzheimer, Parkinson, asthma, diabetes, and obesity (27).

A few studies of pesticide monitoring have been carried out in China to assess the environmental load of pesticide residues and to assess the risk of short-term ingestion of pesticide residues in crops. Fungicides and other pesticide residues in local vegetables were detected in Zhejiang (28) and Shaanxi (29). Another study was conducted in 2018 to investigate the accumulation of pesticide residues in vegetables in 12 Chinese provinces (30), the results showed that 41.9% of the samples were positive. As a large agricultural province with a large population of more than 90 million people (31), Henan Province consumes a great quantity of vegetables, but few studies have detected pesticide residue concentration in vegetables in Henan. In order to ensure food safety and provide a basis for food supervision and regulation, we aimed to assess the pesticide residues of vegetables in Henan Province by using GC-MS method.

## Materials and Methods

### Sampling

**Figure 1:**
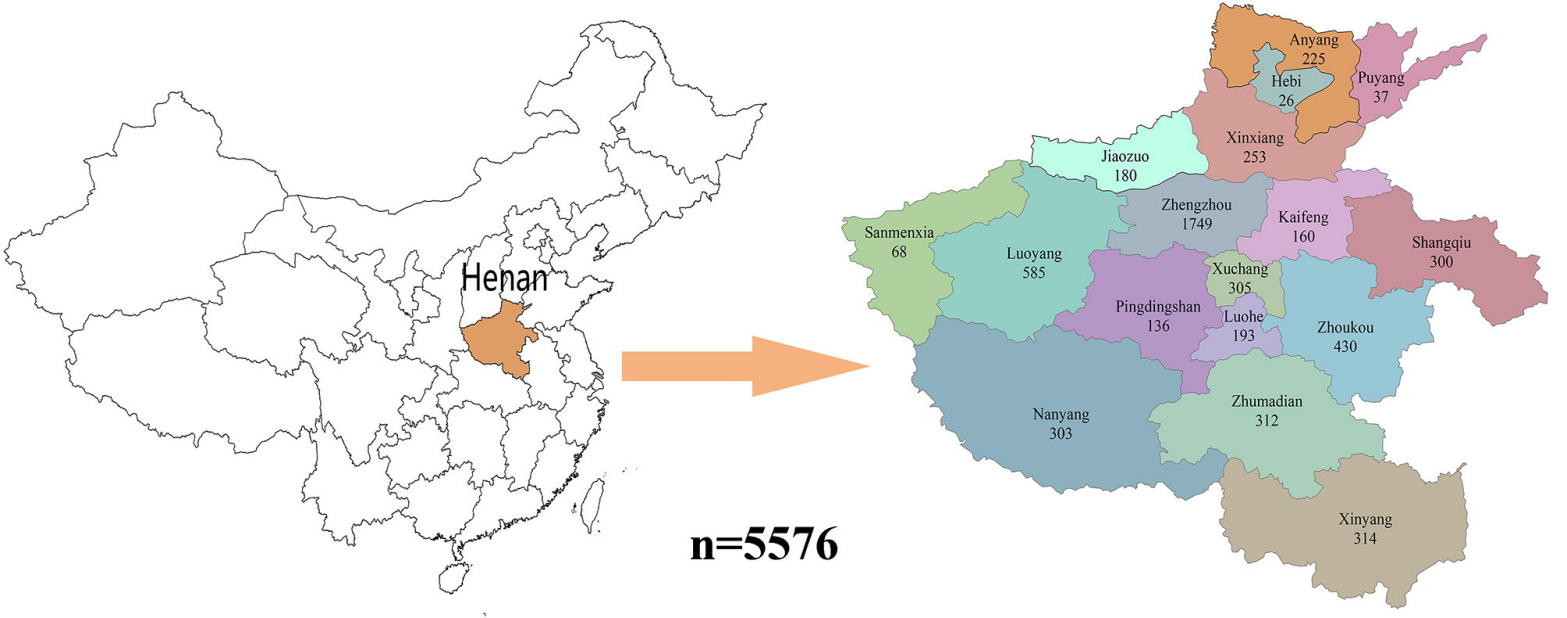
The location and the number of collected samples in each area in Henan.

The locations and the number of samples collected were shown in Figure 1. In this study, a wide variety of vegetables have been selected to detect the pesticide residue, including legumes (green bean and cowpea), root vegetables (ginger and yam), melon vegetables (cucumber), bulb vegetables (leek), eggplant vegetables (tomato, pepper, eggplant, and sweet pepper), leafy vegetables (spinach, common cabbage, celery, and lettuce), and fresh edible mushroom. Most of these vegetables were bought from supermarkets and farmers' markets, with a small proportion of samples coming from 15 locations such as vegetable markets, school canteens, shopping malls, and collective meal delivery units. These vegetables were randomly sampled from 17 prefecture-level administrative regions in Henan Province. A total of 3,234 of these vegetables were obtained from urban areas, while 2,342 samples were selected from rural areas.

### Sample Treatment and Detection

The sample volume for vegetables was in accordance with GB/T 8855, while the edible mushroom samples were randomly sampled at 1 kg and the sampling area was based on GB 2763. For small samples, after sampling, unified treatment. For larger, basically homogeneous samples, they were divided or cut into smaller pieces on the symmetry axis or symmetry plane and then processed. For slender, flat samples or samples with different component contents, they were cut into small pieces or sections in different parts for post-processing. Before sending the samples pre-treated, we have crushed and sieved the samples. Chopped the samples, and then mixed them thoroughly, divided them by quartering or put them directly into a tissue masher to form a homogenate, and then placed them in a polyethylene bottle. According to the national standard GB 23200.113–2018, the sample was first extracted by using acetonitrile, then, the solid-phase extraction method was used to purify and concentrate the samples synchronously. Last, GC-MS was used to conduct qualitative and quantitative analyses. The types of the eight kinds of pesticides tested were procymidone, lambda-cyhalothrin, cypermethrin, pendimethalin, isocarbophos, isazophos, fenthion, and deltamethrin. The detailed steps were as follows.

The samples were firstly pre-treated, and 20 g was transferred into a 100 ml plastic centrifuge tube, adding 40 ml of acetonitrile, homogenizing for 2 min with a high-speed homogeniser at 15,000 revolutions per minute (r/min), and then 5–7 g of sodium chloride was added and shaken vigorously for several times and centrifuged for 5 min at 4200 r/min. The supernatant was sucked into a 100 ml flask, rotary evaporated to 1 ml in a water bath at 40°C, nitrogen blowing until nearly dry. The sample was then purified and washed several times with 3 ml of acetonitrile-toluene solution into a solid phase extraction column. The supernatant was collected, and concentrated in a water bath at 40°C. Then the sample was first re-dissolved with 2.5 ml of ethyl acetate and then added to the internal standard solution for determination. Retention time was determined qualitatively and peak height and peak area were quantified.

### Statistical Analysis

The statistical analysis was performed using IBM SPSS Version 26.0, and *P* < 0.05 was considered statistical significance. Comparison of the detection situation of pesticide residues was performed in different regions by using the chi-square test.

## Results

### The Distributions of Eight Kinds of Pesticides in Different Vegetables

A total of 8 pesticide residues were detected in 5,576 samples in this study. The distributions of eight kinds of pesticides in different vegetables were shown in Table 1. The concentrations of pesticides in some vegetables with high detection rates was showed in Supplementary Figure 1. The 95th percentile (*P*_95_) of detection concentrations in vegetables as follows. Procymidone was detected in leek, cucumber, eggplant, and pepper. In addition, the concentrations of *P*_95_ for leek, cucumber, and eggplant were 3.853, 0.060, and 0.051 (mg/kg), respectively. Lambda-cyhalothrin was detected in 13 kinds of vegetables, and the concentrations of *P*_95_ in leek, celery, tomato, cowpea, lettuce, green bean, and pepper were 0.190, 0.111, 0.062, 0.043, 0.407, 0.034, and 0.048 (mg/kg), respectively. Cypermethrin was detected in 10 kinds of vegetables, and concentrations of *P*_95_ for leek, celery, tomato, common cabbage were 0.439, 0.027, 0.039, and 0.054 (mg/kg), respectively. Few vegetables had a value for the 75th percentile of detection concentrations, such as 0.084 mg/kg procymidone in leek and 0.126 mg/kg cypermethrin in Spinach. The detection rates of pesticide residues in all vegetables were 10.7% and 98.9% of the samples were qualified, samples fail to qualify was leek. Procymidone, lambda-cyhalothrin, cypermethrin, pendimethalin, and isocarbophos were detected in vegetables, the detection rate was 27.0%, 16.2%, 11.4%, 3.5%, and 1.9%, respectively. Isazophos, fenthion and deltamethrin were not detected.

**Table 1:** Description of the detection rate and concentration of various pesticides in each vegetable.

	**No. of samples (n)**	**Detection rate (%)**	**Concentration (mg/kg)**						
			***P*_5_**	***P*_10_**	***P*_25_**	***P*_50_**	***P*_75_**	***P*_90_**	***P*_95_**
**Procymidone**	599								
Leek	452	32.74	< LOD	< LOD	< LOD	< LOD	**0.084**	**1.734**	**3.853**
Cucumber	68	10.29	< LOD	< LOD	< LOD	< LOD	< LOD	**0.014**	**0.060**
Eggplant	39	17.95	< LOD	< LOD	< LOD	< LOD	< LOD	**0.034**	**0.051**
Pepper	40	0	< LOD	< LOD	< LOD	< LOD	< LOD	< LOD	< LOD
**Lambda-cyhalothrin**	1534								
Leek	422	23.46	< LOD	< LOD	< LOD	< LOD	< LOD	**0.071**	**0.190**
Celery	352	16.48	< LOD	< LOD	< LOD	< LOD	< LOD	**0.037**	**0.111**
Tomato	184	17.93	< LOD	< LOD	< LOD	< LOD	< LOD	**0.020**	**0.062**
Cowpea	148	9.46	< LOD	< LOD	< LOD	< LOD	< LOD	< LOD	**0.043**
Lettuce	123	21.10	< LOD	< LOD	< LOD	< LOD	< LOD	**0.244**	**0.407**
Common cabbage	14	7.14	< LOD	< LOD	< LOD	< LOD	< LOD	< LOD	< LOD
Green bean	76	13.16	< LOD	< LOD	< LOD	< LOD	< LOD	**0.020**	**0.034**
Cucumber	68	2.94	< LOD	< LOD	< LOD	< LOD	< LOD	< LOD	< LOD
Pepper	28	7.14	< LOD	< LOD	< LOD	< LOD	< LOD	< LOD	**0.048**
Ginger	50	0	< LOD	< LOD	< LOD	< LOD	< LOD	< LOD	< LOD
Yam	15	0	< LOD	< LOD	< LOD	< LOD	< LOD	< LOD	< LOD
Sweet Pepper	10	0	< LOD	< LOD	< LOD	< LOD	< LOD	< LOD	< LOD
Fresh edible mushroom	44	4.54	< LOD	< LOD	< LOD	< LOD	< LOD	< LOD	< LOD
**Cypermethrin**	1368								
Leek	421	22.8	< LOD	< LOD	< LOD	< LOD	< LOD	**0.184**	**0.439**
Celery	369	7.31	< LOD	< LOD	< LOD	< LOD	< LOD	< LOD	**0.028**
Tomato	151	9.93	< LOD	< LOD	< LOD	< LOD	< LOD	< LOD	**0.039**
Cowpea	148	3.38	< LOD	< LOD	< LOD	< LOD	< LOD	< LOD	**0.005**
Common cabbage	84	7.14	< LOD	< LOD	< LOD	< LOD	< LOD	< LOD	**0.054**
Eggplant	30	0	< LOD	< LOD	< LOD	< LOD	< LOD	< LOD	< LOD
Pepper	68	5.88	< LOD	< LOD	< LOD	< LOD	< LOD	< LOD	< LOD
Ginger	50	0	< LOD	< LOD	< LOD	< LOD	< LOD	< LOD	< LOD
Fresh edible mushroom	42	2.38	< LOD	< LOD	< LOD	< LOD	< LOD	< LOD	< LOD
Spinach	5	40	< LOD	< LOD	< LOD	< LOD	**0.126**	NA	NA
**Pendimethalin**	839								
Leek	451	2.66	< LOD	< LOD	< LOD	< LOD	< LOD	< LOD	< LOD
Celery	388	4.38	< LOD	< LOD	< LOD	< LOD	< LOD	< LOD	< LOD
**Isocarbophos**	216								
Cowpea	216	1.85	< LOD	< LOD	< LOD	< LOD	< LOD	< LOD	< LOD
**Isazophos**	481								
Cowpea	185	0	< LOD	< LOD	< LOD	< LOD	< LOD	< LOD	< LOD
Lettuce	163	0	< LOD	< LOD	< LOD	< LOD	< LOD	< LOD	< LOD
Eggplant	95	0	< LOD	< LOD	< LOD	< LOD	< LOD	< LOD	< LOD
Pepper	28	0	< LOD	< LOD	< LOD	< LOD	< LOD	< LOD	< LOD
Sweet Pepper	10	0	< LOD	< LOD	< LOD	< LOD	< LOD	< LOD	< LOD
**Fenthion**	298								
Cowpea	185	0	< LOD	< LOD	< LOD	< LOD	< LOD	< LOD	< LOD
Green bean	113	0	< LOD	< LOD	< LOD	< LOD	< LOD	< LOD	< LOD
**Deltamethrin**	241								
Tomato	151	0	< LOD	< LOD	< LOD	< LOD	< LOD	< LOD	< LOD
Common cabbage	14	0	< LOD	< LOD	< LOD	< LOD	< LOD	< LOD	< LOD
Green bean	76	0	< LOD	< LOD	< LOD	< LOD	< LOD	< LOD	< LOD

*NA, Not applicable. Bold values indicates detection limit*.

### Description of the Detected Concentrations and Comparison of Detection Rates of Various Pesticides in Contrysides and Cities

As shown in Tables 2, 3, all vegetables were stratified by rural and urban areas to describe the concentrations of pesticides detected in vegetables. It found that procymidone, lambda-cyhalothrin, and cypermethrin were detected in urban areas, while pendimethalin was also detected in rural areas in the countryside. Procymidone was detected in leek, cucumber, and eggplant. Lambda-cyhalothrin was detected in leek, celery, tomato, common cabbage, and pepper. For some of the vegetables with higher detection rates in rural and urban areas, a description of their percentile was shown in Supplementary Figures 2, 3. Among these vegetables, the residue concentrations of the two pesticides in leek were still the highest, the concentrations of *P*_95_ were 2.307 and 0.699 (mg/kg), respectively. The highest concentrations of cypermethrin in these vegetables were detected in lettuce, and the concentration of *P*_95_ was 1.514 mg/kg. Only celery was detected for pendimethalin, and the concentration of *P*_95_ was 0.017 mg/kg. In the city, it was observed that the concentrations of *P*_95_ for leek were the highest in procymidone and cypermethrin, which were 4.596 and 0.134 (mg/kg), respectively. However, common cabbage, not leek, had the highest concentration of lambda-cyhalothrin, which the concentration of *P*_95_ was 1.103 mg/kg.

**Table 2:** Description of the concentrations of various pesticides detected in each vegetable in the countryside.

	**No. of samples (*n*)**	**Concentration (mg/kg)**						
		***P*_5_**	***P*_10_**	***P*_25_**	***P*_50_**	***P*_75_**	***P*_90_**	***P*_95_**
**Procymidone**	203							
Leek	160	< LOD	< LOD	< LOD	< LOD	< LOD	**0.180**	**2.307**
Cucumber	15	< LOD	< LOD	< LOD	< LOD	< LOD	**0.037**	NA
Eggplant	14	< LOD	< LOD	< LOD	< LOD	**0.033**	**0.073**	NA
Pepper	14	< LOD	< LOD	< LOD	< LOD	< LOD	< LOD	< LOD
**Lambda-cyhalothrin**	580							
Leek	145	< LOD	< LOD	< LOD	< LOD	< LOD	**0.388**	**0.699**
Celery	193	< LOD	< LOD	< LOD	< LOD	< LOD	< LOD	**0.034**
Tomato	71	< LOD	< LOD	< LOD	< LOD	< LOD	**0.021**	**0.047**
Cowpea	74	< LOD	< LOD	< LOD	< LOD	< LOD	< LOD	< LOD
Common cabbage	43	< LOD	< LOD	< LOD	< LOD	< LOD	< LOD	**0.054**
Eggplant	7	< LOD	< LOD	< LOD	< LOD	< LOD	< LOD	< LOD
Pepper	20	< LOD	< LOD	< LOD	< LOD	< LOD	< LOD	**0.017**
Ginger	17	< LOD	< LOD	< LOD	< LOD	< LOD	< LOD	< LOD
Fresh edible mushroom	10	< LOD	< LOD	< LOD	< LOD	< LOD	< LOD	< LOD
**Cypermethrin**	635							
Leek	145	< LOD	< LOD	< LOD	< LOD	**0.019**	**0.142**	**0.257**
Celery	185	< LOD	< LOD	< LOD	< LOD	< LOD	**0.072**	**0.178**
Tomato	83	< LOD	< LOD	< LOD	< LOD	< LOD	**0.030**	**0.081**
Cowpea	74	< LOD	< LOD	< LOD	< LOD	< LOD	**0.032**	**0.065**
Lettuce	58	< LOD	< LOD	< LOD	< LOD	**0.020**	**0.383**	**1.514**
Common cabbage	3	< LOD	< LOD	< LOD	< LOD	< LOD	< LOD	< LOD
Green bean	28	< LOD	< LOD	< LOD	< LOD	< LOD	**0.034**	**0.044**
Cucumber	15	< LOD	< LOD	< LOD	< LOD	< LOD	< LOD	< LOD
Pepper	6	< LOD	< LOD	< LOD	< LOD	< LOD	< LOD	< LOD
Ginger	17	< LOD	< LOD	< LOD	< LOD	< LOD	< LOD	< LOD
Yam	9	< LOD	< LOD	< LOD	< LOD	< LOD	< LOD	< LOD
Fresh edible mushroom	11	< LOD	< LOD	< LOD	< LOD	< LOD	< LOD	< LOD
**Pendimethalin**	367							
Leek	160	< LOD	< LOD	< LOD	< LOD	< LOD	< LOD	< LOD
Celery	207	< LOD	< LOD	< LOD	< LOD	< LOD	< LOD	**0.017**
**Isocarbophos**	107							
Cowpea	107	< LOD	< LOD	< LOD	< LOD	< LOD	< LOD	< LOD
**Isazophos**	211							
Cowpea	98	< LOD	< LOD	< LOD	< LOD	< LOD	< LOD	< LOD
Lettuce	73	< LOD	< LOD	< LOD	< LOD	< LOD	< LOD	< LOD
Eggplant	33	< LOD	< LOD	< LOD	< LOD	< LOD	< LOD	< LOD
Pepper	6	< LOD	< LOD	< LOD	< LOD	< LOD	< LOD	< LOD
Sweet Pepper	1	< LOD	< LOD	< LOD	< LOD	< LOD	< LOD	< LOD
**Fenthion**	137							
Cowpea	98	< LOD	< LOD	< LOD	< LOD	< LOD	< LOD	< LOD
Green bean	39	< LOD	< LOD	< LOD	< LOD	< LOD	< LOD	< LOD
**Deltamethrin**	102							
Tomato	71	< LOD	< LOD	< LOD	< LOD	< LOD	< LOD	< LOD
Common cabbage	3	< LOD	< LOD	< LOD	< LOD	< LOD	< LOD	< LOD
Green bean	28	< LOD	< LOD	< LOD	< LOD	< LOD	< LOD	< LOD

*NA, Not applicable. Bold values indicates detection limit*.

**Table 3:** Description of the concentrations of various pesticides detected in each vegetable in the city.

	**No. of samples (*n*)**	**Concentration (mg/kg)**						
		***P*_5_**	***P*_10_**	***P*_25_**	***P*_50_**	***P*_75_**	***P*_90_**	***P*_95_**
**Procymidone**	396							
Leek	292	< LOD	< LOD	< LOD	< LOD	**0.118**	**2.255**	**4.596**
Cucumber	53	< LOD	< LOD	< LOD	< LOD	< LOD	**0.009**	**0.064**
Eggplant	25	< LOD	< LOD	< LOD	< LOD	< LOD	< LOD	**0.037**
Pepper	26	< LOD	< LOD	< LOD	< LOD	< LOD	< LOD	< LOD
**Lambda-cyhalothrin**	788							
Leek	276	< LOD	< LOD	< LOD	< LOD	< LOD	**0.102**	**0.372**
Celery	176	< LOD	< LOD	< LOD	< LOD	< LOD	< LOD	**0.048**
Tomato	80	< LOD	< LOD	< LOD	< LOD	< LOD	< LOD	**0.055**
Cowpea	74	< LOD	< LOD	< LOD	< LOD	< LOD	< LOD	**0.018**
Common cabbage	41	< LOD	< LOD	< LOD	< LOD	< LOD	< LOD	**1.103**
Eggplant	23	< LOD	< LOD	< LOD	< LOD	< LOD	< LOD	< LOD
Pepper	48	< LOD	< LOD	< LOD	< LOD	< LOD	< LOD	**0.024**
Ginger	33	< LOD	< LOD	< LOD	< LOD	< LOD	< LOD	< LOD
Fresh edible mushroom	32	< LOD	< LOD	< LOD	< LOD	< LOD	< LOD	0.038
Spinach	5	< LOD	< LOD	< LOD	< LOD	**0.126**	NA	NA
**Cypermethrin**	899							
Leek	277	< LOD	< LOD	< LOD	< LOD	< LOD	**0.049**	**0.134**
Celery	167	< LOD	< LOD	< LOD	< LOD	< LOD	**0.016**	**0.076**
Tomato	101	< LOD	< LOD	< LOD	< LOD	< LOD	< LOD	**0.053**
Cowpea	74	< LOD	< LOD	< LOD	< LOD	< LOD	< LOD	**0.018**
Lettuce	65	< LOD	< LOD	< LOD	< LOD	< LOD	**0.105**	**0.350**
Common cabbage	11	< LOD	< LOD	< LOD	< LOD	< LOD	< LOD	< LOD
Green bean	48	< LOD	< LOD	< LOD	< LOD	< LOD	< LOD	**0.019**
Cucumber	53	< LOD	< LOD	< LOD	< LOD	< LOD	< LOD	< LOD
Pepper	22	< LOD	< LOD	< LOD	< LOD	< LOD	< LOD	**0.065**
Ginger	33	< LOD	< LOD	< LOD	< LOD	< LOD	< LOD	< LOD
Yam	6	< LOD	< LOD	< LOD	< LOD	< LOD	< LOD	< LOD
Sweet Pepper	9	< LOD	< LOD	< LOD	< LOD	< LOD	< LOD	< LOD
Fresh edible mushroom	33	< LOD	< LOD	< LOD	< LOD	< LOD	< LOD	0.011
**Pendimethalin**	472							
Leek	291	< LOD	< LOD	< LOD	< LOD	< LOD	< LOD	< LOD
Celery	181	< LOD	< LOD	< LOD	< LOD	< LOD	< LOD	< LOD
**Isocarbophos**	109	< LOD	< LOD	< LOD	< LOD	< LOD	< LOD	< LOD
Cowpea	109	< LOD	< LOD	< LOD	< LOD	< LOD	< LOD	< LOD
**Isazophos**	270	< LOD	< LOD	< LOD	< LOD	< LOD	< LOD	< LOD
Cowpea	87	< LOD	< LOD	< LOD	< LOD	< LOD	< LOD	< LOD
Lettuce	90	< LOD	< LOD	< LOD	< LOD	< LOD	< LOD	< LOD
Eggplant	62	< LOD	< LOD	< LOD	< LOD	< LOD	< LOD	< LOD
Pepper	22	< LOD	< LOD	< LOD	< LOD	< LOD	< LOD	< LOD
Sweet Pepper	9	< LOD	< LOD	< LOD	< LOD	< LOD	< LOD	< LOD
**Fenthion**	161	< LOD	< LOD	< LOD	< LOD	< LOD	< LOD	< LOD
Cowpea	87	< LOD	< LOD	< LOD	< LOD	< LOD	< LOD	< LOD
Green bean	74	< LOD	< LOD	< LOD	< LOD	< LOD	< LOD	< LOD
**Deltamethrin**	139	< LOD	< LOD	< LOD	< LOD	< LOD	< LOD	< LOD
Tomato	80	< LOD	< LOD	< LOD	< LOD	< LOD	< LOD	< LOD
Common cabbage	11	< LOD	< LOD	< LOD	< LOD	< LOD	< LOD	< LOD
Green bean	48	< LOD	< LOD	< LOD	< LOD	< LOD	< LOD	< LOD

*NA, Not applicable. Bold values indicates detection limit*.

The detection rates of procymidone, lambda-cyhalothrin and isocarbophos in urban areas were 29.3%, 12.2%, and 2.8%, respectively, which were higher than rural areas (22.7%, 10.3%, and 0.9%). No significant difference in pesticide residues was observed between the two groups. In contrast, the detection rates of cypermethrin and pendimethalin in rural were 19.8% and 5.4%, respectively, which were higher than urban vegetables (13.7% and 1.9%) (*P* < 0.05). The detailed results were shown in Table 4.

**Table 4:** Comparison of detection rates of various pesticides between rural and city.

**Pesticide**	**No. of samples (n)**		**Detection rate (%)**		***P***
	**countryside**	**city**	**countryside**	**city**	
Procymidone	203	396	22.7	29.3	0.084
Cypermethrin	635	899	19.8	13.7	**0.001**
Lambda-cyhalothrin	580	788	10.3	12.2	0.291
Pendimethalin	367	472	5.4	1.9	**0.005**
Isocarbophos	107	109	0.9	2.8	0.322
Isazophos	211	270	0	0	NA
Fenthion	137	161	0	0	NA
Deltamethrin	102	139	0	0	NA

*NA, Not applicable. Bold values indicates detection limit*.

### Pesticide Residues in Different Areas

Based on topography and rivers, the Henan province was divided into five parts: east, west, south, north, and central. The detection rates of procymidone, cypermethrin, and lambda-cyhalothrin resident in the eastern part of Henan Province were the highest, and the corresponding detection rates were 38.0%, 21.5%, and 18.8%, respectively. The lowest residue level of procymidone, cypermethrin and lambda-cyhalothrin in vegetables was measured in western, southern, northern Henan and the corresponding detection rates were 10.9%, 13.5%, and 8.2%, respectively, as shown in Table 5. And there were statistical differences in the detection rates of procymidone, cypermethrin and lambda-cyhalothrin in different regions (*P* < 0.05).

**Table 5:** Detection rate of various pesticides in different areas of Henan province.

**Pesticide**	**No. of samples (n) Detection rate**					***P***
	**Eastern Henan**	**Western Henan**	**Southern Henan**	**Northern Henan**	**Central subregion of Henan**	
Procymidone	79(38%)	119(10.9%)	86(22.1%)	77(14.3%)	238(37.4%)	**0.000**
Cypermethrin	251(21.5%)	200(20.5%)	251(13.5%)	190(14.7%)	642(14.0%)	**0.019**
Lambda-cyhalothrin	213(18.8%)	188(10.1%)	220(8.2%)	174(6.9%)	573(11.7%)	**0.001**
Pendimethalin	142(3.5%)	95(0%)	138(2.2%)	87(4.6%)	377(4.5%)	0.225
Isocarbophos	34(0%)	33(0%)	44(2.3%)	40(42.5%)	65(3.1%)	0.747
Isazophos	87(0%)	76(0%)	94(0%)	73(0%)	151(0%)	NA
Fenthion	46(0%)	40(0%)	63(0%)	47(0%)	102(0%)	NA
Deltamethrin	38(0%)	38(0%)	33(0%)	33(0%)	99(0%)	NA

*NA, Not applicable. Bold values indicates detection limit*.

## Discussion

A total of 10.7% samples were detected pesticide residues. In another region of China and some developing countries (32–35), a high incidence of pesticide residues was found. Among all the samples tested, there was a higher detection rate of procymidone, lambda-cyhalothrin and cypermethrin. Similar results were obtained for samples tested in the Aegean region of Turkey (10). In our detection process, leek, celery, tomato, cowpea, and pepper were found to have higher detection rates as they may be more susceptible to insect pests. However, the types of pesticide residues were not exactly the same for each vegetable, indicating the irregular distribution of various pesticides in each vegetable as well as the different pesticide application habits of vegetable farmers (36).

After stratified analysis, we found that the detection rates of lambda-cyhalothrin and pendimethalin were higher in rural than urban areas, and this difference was statistically significant. In a survey conducted in a rural French community, it was concluded that pendimethalin was the most commonly measured pesticide when investigating local atmospheric pesticide levels during pesticide application (37), which is consistent with the results of our study, probably because pendimethalin is widely used in rural agriculture due to its wide range of applications and low toxicity (38), and some farmers cannot use it correctly. In future agricultural education, it is important to enhance the knowledge of the use of pesticides so that every farmer can follow Good Agricultural Practices (GAP) (39).

A comparison of the detection rates of pesticides in several regions in Henan revealed that the detection rates of procymidone, lambda-cyhalothrin, and cypermethrin were higher in eastern Henan than other regions, and this difference was statistically significant. This result may be due to the fact that the economic pillar of Eastern Henan has always been agriculture, being the most important grain-producing area in Henan. The level of economic development is an important contributor to pesticide residue (40). Compared to other areas in Henan, the industrial base is poor and the lack of certain research institutes leads to a weaker cultural knowledge of the local people, which also has an impact on the use of pesticides, and it is urgent to strengthen the education and management of farmers.

As we know, few studies were conducted to determine the levels of pesticide residues on various vegetables frequently consumed in Henan province. In China, pesticides are widely used in crops, and a total of 13.1 kg per hectare was used per unit of cropland averagely (41), especially in vegetables (9), so we detect several pesticide residues commonly found in vegetables. Although a large proportion of the samples were not detected for pesticides, we still need to put emphasis on food safety. In future research, we need to pay more attention to what factors influence pesticide residues in vegetables so that we can resolve the problem from source. And we can't ignore the serious health effects of other pollutants on human beings and environmental pollution (42, 43), such as PCB (44) and PAHs (45, 46). In addition, better supervision and regulation of pesticides as well as education of farmers is an important issue.

## Conclusions

The results have revealed that the pesticide residues are present in commonly consumed vegetables in Henan Province of China. The study provides scientific evidences of detected residues of familiar pesticides in the commonly used vegetables in Henan Province. More attention should be paid to these vegetables and areas with high detection rates, to control pesticide residues at source and ensure food safety for residents.

## Data Availability Statement

The raw data supporting the conclusions of this article will be made available by the authors, without undue reservation.

## Author Contributions

Material preparation and data analysis were performed by CM and DW. The first draft of the manuscript was written by CM and all authors commented on previous versions of the manuscript. All authors contributed to the article and approved the submitted version.

## Funding

This research was supported by the National Key Research and Development Program of China (Grant No: 2019YFC1710002), the National Natural Science Foundation of China (Grant Nos: 42177415 and 21806146), the Postdoctoral Science Foundation of China (Grant Nos: 2020T130604 and 2021M702934), the Science and Technique Foundation of Henan Province (Grant No: 212102310074), the Scientific and Technological Innovation of Colleges and Universities in Henan Province Talent Support Program (Grant No: 22HASTIT044), the Young Backbone Teachers Program of Colleges and Universities in Henan Province (Grant No: 2021GGJS015), and the Excellent Youth Development Foundation of Zhengzhou University (Grant No: 2021ZDGGJS057).

## Conflict of Interest

The authors declare that the research was conducted in the absence of any commercial or financial relationships that could be construed as a potential conflict of interest.

## Publisher's Note

All claims expressed in this article are solely those of the authors and do not necessarily represent those of their affiliated organizations, or those of the publisher, the editors and the reviewers. Any product that may be evaluated in this article, or claim that may be made by its manufacturer, is not guaranteed or endorsed by the publisher.

## Supplementary Material

Supplementary Figure 1 Concentrations of pesticides in some vegetables with high detection rates.

Supplementary Figure 2 Concentrations of pesticides in some vegetables with high detection rates in the countryside.

Supplementary Figure 3 Concentrations of pesticides in some vegetables with high detection rates in the city.

## Abbreviations

GC-MS: gas chromatography-mass spectrometry
r/min: revolutions per minute.
