# Supplementary figures and images for: Pesticide Residues in Commonly Consumed Vegetables in Henan Province of China in 2020
Source: Front Public Health. 2022 Jun 10;10:901485. doi: 10.3389/fpubh.2022.901485 (PMC9226416; doi:10.3389/fpubh.2022.901485)

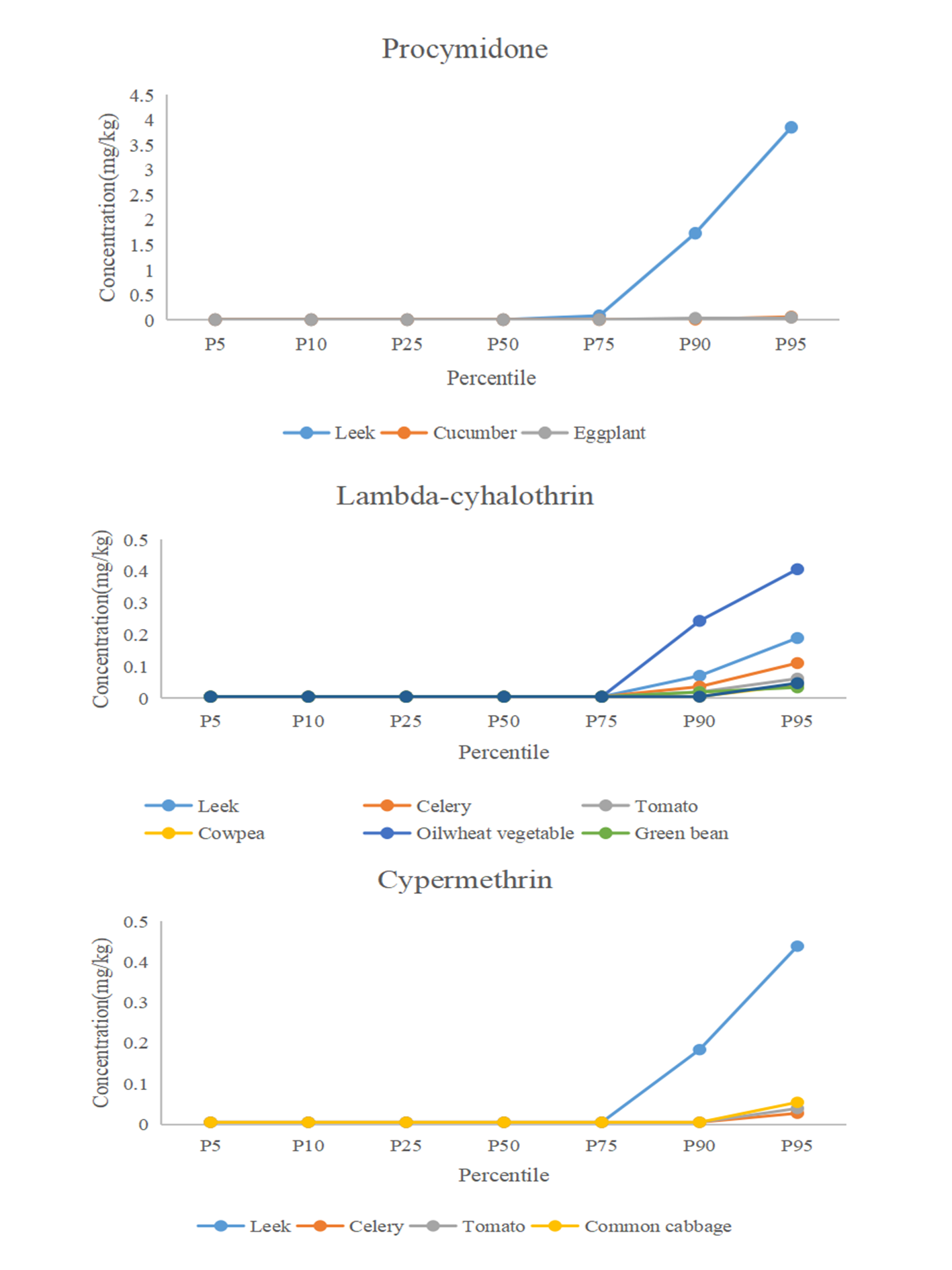

Supplement: Supplementary Figure 1 — Concentrations of pesticides in some vegetables with high detection rates. [file Image_1.TIF]

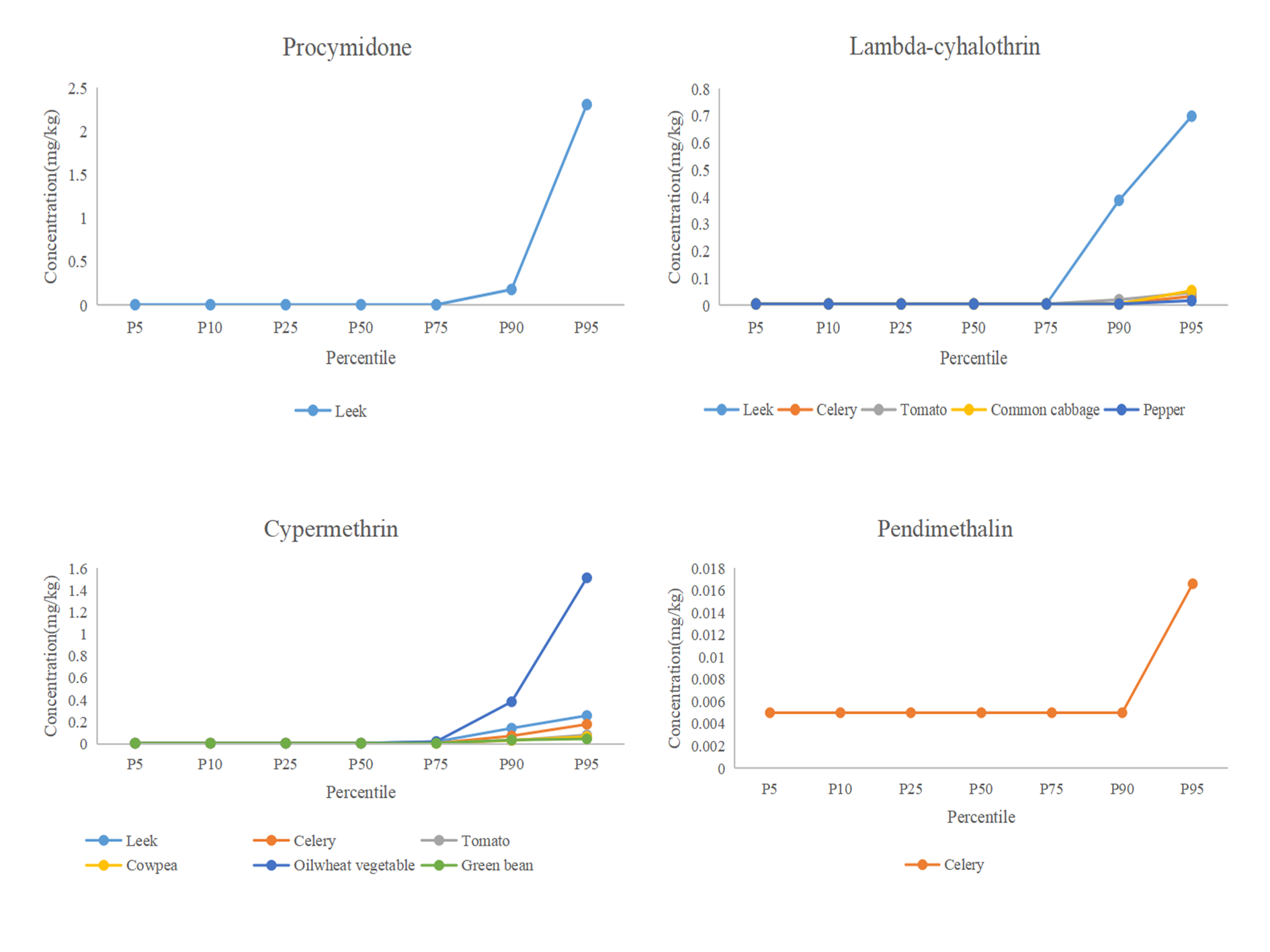

Supplement: Supplementary Figure 2 — Concentrations of pesticides in some vegetables with high detection rates in the countryside. [file Image_2.TIF]

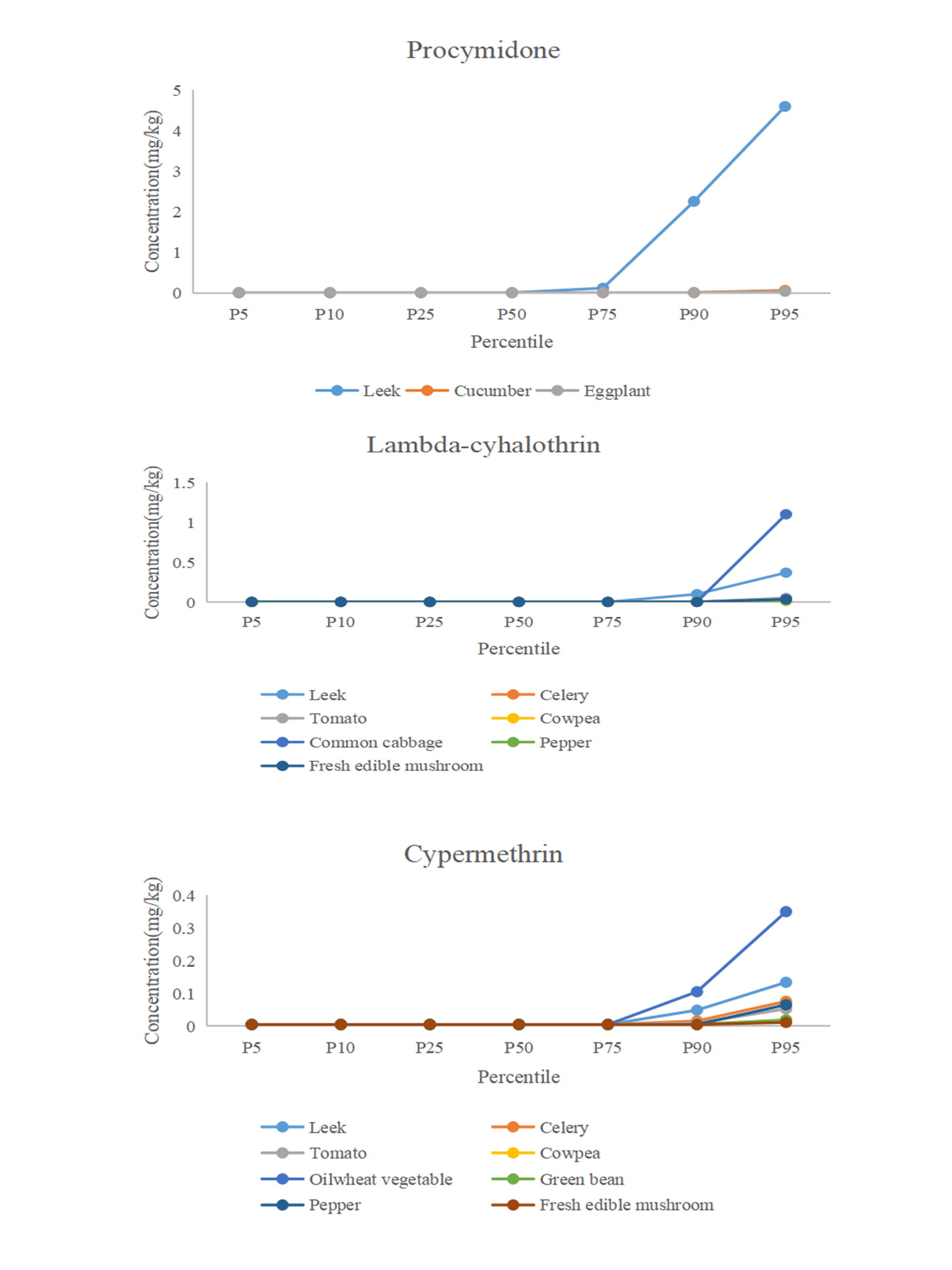

Supplement: Supplementary Figure 3 — Concentrations of pesticides in some vegetables with high detection rates in the city. [file Image_3.TIF]
